# News and Notes

**Published:** 1910-08

**Authors:** 


### REGULAR MEETING OF THE FULTON COUNTY MEDICAL SOCIETY.

### JUNE 16TH., 1910.

REPORTED BY R. R. DALY, M. D.

Dr. Frank K. Boland read a paper on "More Care in the Diagnosis and Treatment of Fractures."

Dr. Andrews said he agreed as a rule with Dr. Boland, but he could not concur in the open operation in the reduction of the fractures of the femur. There is an apparatus, which, if applied under an anaesthetic, making a plaster spica from toes to shoulder, will give good results. In the fracture of the humerus, it should be applied from the shoulder to the fingers. Plaster-of-Paris, as a rule if properly applied, will give good results.

Dr. Hancock said that splints were needed at the elbows but the ordinary straight ones will not hold round about.

Plaster is used here. The olecranon should be opened and wired. The X-Ray is deceptive in these cases even with the best photographs. In fractures high in the radius he prefers the circular casts in pronation. In high femur fractures he uses plaster casts with extension and the knee and hip flexed. The public is pretty well educated to the deformity of this accident and expect it to follow, but that is no reason why we should not make every effort to do away with it.

Dr. Boland said, in closing, that this subject was not given enough attention because of its general dryness; osteology always seems so. He has recently seen three fractures of the neck of the femur in old people, that were neglected and were followed by bed sores and pneumonia, etc.

Dr. Strickler was not present to read his paper.

Dr. Andrews said in discussing auto-intoxication that the first line of defense was in the intestines themselves. Here we find toxines, colloidal substances and albumoses. If the intestinal mucosa is intact it takes five times as much poison to produce death as when it is injured. The second defense is in the liver. Indol, phenol, skatol and cresol are taken from the blood by the liver, made into various sulphates, etc., and ultimately are secreted by the kidneys. The prenchyma of the liver has an affinity for cresol and phenol; ammoniacal substances are changed into urea; purin and xanthin bases are changed into uric acid; alkaloids are destroyed in the liver as well as alcohol. Any hepatic insufficiency would of itself, give auto-intoxication. The ductless glands play an important part in taking out poisons. Thyrevidectomy in carnivorae invariably causes death. The suprenal glands neutralize nicotine.

Thompson & Kemp have shown that the minor epilepsies are often relieved by curing auto-intoxication. Equally is this true of certain affections of the bones and joints. Dr. Andrews now has the case of a child with decalcification of the bone due to intense infection of the intestines. There are many more remote things caused by this condition. Intestinal flora are aerobic naturally and the putrefaction germs gain admission and putrefy albumens. The treatment lies in the cessation of animal albumens in the food, the physical removal of the putrefaction germs by high colon injections, the introduction of antiseptics and the giving of lactic acid bacilli (Metchnikoff) in the food. These are hard to introduce permanently.

Dr. Adair said he had had auto-intoxication himself and knows what it means. There is no doubt that anti-bacteria are of value in combatting this trouble. Slow, regular assimilation of these toxins cause premature senility. A large per cent of the neurasthenics have auto-intoxication; it is this influence that complicates genito-urinary diseases. In his work as a dentist he finds that Riggs disease causes auto-intoxication rather then the reverse. Pus is flowing constantly into the mouth and stomach at the rate of a drachm per hour and there is sure to be the indicanurea. Cleansing the mouth in these cases frequently clears the indican.

Dr. George H. Noble read a paper on "The Use of the Rubber Dam in Gastro-Enterostomy and Intestinal Anastamoses" which will be published elsewhere in the Journal.

Dr. Goldsmith said it was an excellent device and that Dr. Noble should. claim a distinct advance in the technique of abdominal surgery.

Dr. C. P. Ward read a paper on "Two Cases of Tentanus Treated with Spirits of Turpentine."

Dr. Goldsmith said the report of two cases cured was worth considering. His own cases have died usually with various treatments of serum, etc. He now has a case of spasm of the jaws after enucleation of the eye. Chlorotone was used by some one in Detroit and has cured several cases. Anything that can cure even one per cent of the cases is doing well. Railroad cases of crushed wounds are almost always saturated with turpentine at the time of the accident by trainmen and, strangely, little or no tentanus follows.

Dr. Andrews said that turpentine is absorbed through the skin and is one of the few drugs that can be so used. It is an excellent antiseptic.

Dr. Noble said it would be of interest if Dr. Ward would go farther and make a laboratory test of turpentine in tentanus. Some of these cases so widely treated might have got well of themselves; he, himself, has had two cases recover under the serum treatment but is by no means sure that the serum is reponsible.

